# Discovery of Molecular DNA Methylation-Based Biomarkers through Genome-Wide Analysis of Response Patterns to BCG for Bladder Cancer

**DOI:** 10.3390/cells9081839

**Published:** 2020-08-05

**Authors:** Dafina Ilijazi, Walter Pulverer, Iris E. Ertl, Ursula Lemberger, Shoji Kimura, Mohammad Abufaraj, David D’Andrea, Benjamin Pradere, Andreas Bruchbacher, Anna Graf, Francesco Soria, Martin Susani, Andrea Haitel, Luca Molinaro, Armin Pycha, Evi Comploj, Stephan Pabinger, Andreas Weinhäusel, Gerda Egger, Shahrokh F. Shariat, Melanie R. Hassler

**Affiliations:** 1 Department of Urology, Medical University of Vienna, 1090 Vienna, Austria; dafinailijazii@gmail.com (D.I.); iris.ertl@meduniwien.ac.at (I.E.E.); ursula.lemberger@meduniwien.ac.at (U.L.); shoji.kimura-1228@hotmail.co.jp (S.K.); dr.abufaraj@gmail.com (M.A.); david.dandrea@meduniwien.ac.at (D.D.); benjaminpradere@gmail.com (B.P.); andreas.bruchbacher@meduniwien.ac.at (A.B.); anna@airmix.at (A.G.); soria.fra@gmail.com (F.S.); 2 AIT—Austrian Institute of Technology GmbH, Health & Environment Department, Molecular Diagnostics, 1210 Vienna, Austria; walter.pulverer@ait.ac.at (W.P.); stephan.pabinger@gmail.com (S.P.); andreas.weinhaeusel@ait.ac.at (A.W.); 3 Department of Urology, Jikei University School of Medicine, Tokyo 105-8461, Japan; 4 Division of Urology, Department of Special Surgery, The University of Jordan, Amman 11942, Jordan; 5 Department of Urology, CHRU Tours, Francois Rabelais University, 37000 Tours, France; 6 Division of Urology, Department of Surgical Sciences, San Giovanni Battista Hospital, University of Studies of Torino, 10124 Turin, Italy; 7 Clinical Institute of Pathology, Medical University of Vienna, Vienna 1090, Austria; martin.susani@meduniwien.ac.at (M.S.); andrea.haitel@meduniwien.ac.at (A.H.); gerda.egger@meduniwien.ac.at (G.E.); 8 Division of Pathology, Department of Medical Sciences, University of Studies of Torino, 10124 Turin, Italy; luca.molinaro@unito.it; 9 Department of Urology, Central Hospital of Bolzano/Bozen, 39100 Bozen, Italy; armin.pycha@sabes.it (A.P.); complojevi94@yahoo.de (E.C.); 10 Sigmund Freud Private University, Medical University, 1020 Vienna, Austria; 11 College of Health-Care Professions, Claudiana Research, Claudiana, 39100 Bolzano, Italy; 12 Ludwig Boltzmann Institute Applied Diagnostics, Währinger Gürtel 18-20, 1090 Vienna, Austria; 13 Department of Urology, University of Texas Southwestern Medical Center, Dallas, TX 75390, USA; 14 Department of Urology, Weill Cornell Medical College, New York, NY 10065, USA; 15 Karl Landsteiner Institute of Urology and Andrology, 3100 St. Poelten, Austria; 16 Department of Urology, Second Faculty of Medicine, Charles University, 150 06 Prague, Czech Republic; 17 Institute for Urology and Reproductive Health, I.M. Sechenov First Moscow State Medical University, 119992 Moscow, Russia; 18 European Association of Urology research foundation, 6842 Arnhem, Netherlands

**Keywords:** bladder cancer, Bacillus Calmette-Guérin, DNA methylation marker, urothelial cancer, Illumina MethylationEPIC BeadChip, high-risk bladder cancer, BCG refractory

## Abstract

Background: Bacillus Calmette-Guérin (BCG) immunotherapy, the standard adjuvant intravesical therapy for some intermediate and most high-risk non-muscle invasive bladder cancers (NMIBCs), suffers from a heterogenous response rate. Molecular markers to help guide responses are scarce and currently not used in the clinical setting. Methods: To identify novel biomarkers and pathways involved in response to BCG immunotherapy, we performed a genome-wide DNA methylation analysis of NMIBCs before BCG therapy. Genome-wide DNA methylation profiles of DNA isolated from tumors of 26 BCG responders and 27 failures were obtained using the Infinium MethylationEPIC BeadChip. Results: Distinct DNA methylation patterns were found by genome-wide analysis in the two groups. Differentially methylated CpG sites were predominantly located in gene promoters and gene bodies associated with bacterial invasion of epithelial cells, chemokine signaling, endocytosis, and focal adhesion. In total, 40 genomic regions with a significant difference in methylation between responders and failures were detected. The differential methylation state of six of these regions, localized in the promoters of the genes *GPR158*, *KLF8*, *C12orf42*, *WDR44*, *FLT1,* and *CHST11,* were internally validated by bisulfite-sequencing. *GPR158* promoter hypermethylation was the best predictor of BCG failure with an AUC of 0.809 (*p*-value < 0.001). Conclusions: Tumors from BCG responders and BCG failures harbor distinct DNA methylation profiles. Differentially methylated DNA regions were detected in genes related to pathways involved in bacterial invasion of cells or focal adhesion. We identified candidate DNA methylation biomarkers that may help to predict patient prognosis after external validation in larger, well-designed cohorts.

## 1. Introduction

Bacillus Calmette-Guérin (BCG) immunotherapy has been effectively used in the last 30 years for the management of non-muscle-invasive bladder cancer (NMIBC). It is, indeed, the standard intravesical adjuvant therapy for intermediate- and high-risk NMIBC, as it prevents/delays tumor recurrence and/or progression [1,2,3]. Complete response is observed in approximately 70% of patients, whereas 30% experience recurrence and progression to a more aggressive disease state [4,5]. Despite clinical familiarity with BCG, its molecular mode of action remains only partially understood. Both benign and cancerous urothelial cells, together with the local immune system, play an active role in BCG’s therapeutic anti-tumor effect. Accurate prediction of BCG response is essential to improve clinical decisions by identifying the patients most likely to respond sustainably and those who are most likely to benefit from alternative strategies while avoiding undesirable side effects of ineffective BCG treatment. Various clinical and molecular biomarkers have been tested to help advance the personalized BCG therapy allocation. On the molecular level, internalization of BCG into urothelial cells induces the production of immune-stimulatory cytokines and chemokines, thereby stimulating several types of inflammatory cells. As a result, the anti-tumor response by the immune-mediated killing of infected malignant cells is enhanced [6,7,8,9,10]. The exact molecular mechanisms leading to cancer cell elimination are still unclear. However, attachment of BCG to the urothelium, interaction with integrins, and bacterial uptake into cancer cells have been identified as critical steps in various in vitro and in vivo studies [11,12,13,14,15,16,17,18,19,20].

Currently, no molecular marker predicting response to BCG therapy is available in clinical routine, but several studies have reported the use of predictive biomarkers for BCG outcome [21]. Recently, the predictive potential of the DNA methylation state of selected CpG sites has been evaluated [22,23]. Among these, to our knowledge, only one study was based on genome-wide DNA methylation profiling of BCG responders and failures [23]. To characterize differences in DNA methylation profiles between BCG responders and failures, we used the Infinium MethylationEPIC BeadChip platform [24], a DNA methylation microarray, which interrogates more than 850,000 CpG sites of the human genome. Our aim was to discover DNA methylation profiles that could help identify the patients most likely to benefit from BCG using the most comprehensive array available as a basis for further research. We show that BCG responders and failures harbor distinct DNA methylation profiles, and DNA regions with differential DNA methylation are present in genes related to bacterial invasion or focal adhesion. We also identify candidate DNA methylation biomarkers for stratification to BCG therapy response.

## 2. Materials and Methods

### 2.1. Human Tissue Samples

Formalin-fixed paraffin-embedded (FFPE) tissues of patients with intermediate or high-risk NMIBC, who had received transurethral resection of the bladder (TURB) prior to BCG immunotherapy at our institution between 1994 and 2014 were included in this retrospective study. Patients were defined as BCG responders when no tumors were detected six to twelve months after BCG treatment. The occurrence of low-grade pTa tumors was not considered as an event [1,25]. Patients experiencing disease progression within three to six months or persistent high-grade disease at six months after starting therapy (BCG refractory), as well as patients with tumor relapse within six months of last BCG exposure (BCG early recurrence), were considered BCG unresponsive. We also included patients suffering from tumor relapse later than six months after the last BCG exposure (late recurrence). All these patients were defined as BCG failures (Figure S1). We identified 26 responders and 27 failures meeting these definition criteria (discovery cohort). FFPE tumor samples from initial TURBs were histopathologically checked to confirm the presence of more than 80% of tumor tissue per sample. Validation of targets showing differential methylation was performed using tissue of patients from the same cohort (internal validation, *n* = 37; responders = 19, failures = 18) and also from an external cohort (*n* = 39; responders = 20, failures = 19); for patient characteristics of the internal and external cohort see Table S1). Approval for performing the study was obtained from the institutional ethics committees (2282/2016).

### 2.2. DNA Extraction and Bisulfite Conversion

DNA was extracted from FFPE tissues using the DNA BLOOD Mini KIT (Qiagen). Samples were incubated with proteinase K (Qiagen, Hilden, Germany) for 72 h at 56 °C and subsequently processed according to the manufacturer’s instructions. DNA concentrations were measured using the Qubit Fluorometric quantification system (Thermo Fisher Scientific, Waltham, MA, USA).

### 2.3. Epigenome-Wide DNA Methylation Analysis with ILLUMINA’S Infinium MethylationEPIC BeadChips

500 ng of the isolated DNA from the discovery cohort (26 responders and 27 failures) were used for bisulfite-conversion using the EZ DNA Methylation Kit (Zymo Research, Irvine, CA, USA) according to the manufacturer’s instructions. In brief, bisulfite conversion was carried out overnight in a thermocycler (95 °C for 30 s; 50 °C for 60 min; 16 cycles). Deaminated DNA was purified and eluted with sterile water. Correct bisulfite conversion was verified by qPCR using 2 µL of the 1:4 diluted eluate, targeting methylated regions of *DNAJC15* and of the *GNAS* locus (one assay for the unmethylated allele and one assay for the methylated allele). Identical amounts of deaminated DNA (20 ng/reaction) isolated from blood was amplified in parallel and served as a positive control. The quality of a respective sample was rated as sufficient when the ct-value either for the two *GNAS* loci or the *DNAJC15* locus reached the threshold not later than five cycles compared to the positive control. Subsequently, the bisulfite-converted DNA was subjected to the Infinium procedure according to the manufacturer’s protocol for the Infinium Assay version 11322371 Rev A. Briefly, deaminated DNA was subjected to genome-wide amplification, following an isothermal incubation step at 37 °C for 20 h. DNA was enzymatically fragmented, purified, and precipitated. The precipitated DNA was resuspended in RA1 buffer, denatured at 95 °C for 20 min, and applied to the BeadChip for hybridization at 48 °C for 20 h. Subsequently, non-hybridized fragments were removed. The data of the presented study were generated in two different batches.

### 2.4. Data and Pathway Analysis

Raw data was extracted from the idat.files using the R environment and the ChAMP package. We replaced missing values by a knn-algorithm included in the ChAMP package and included CpGs located on the XY chromosomes in the analysis [26]. A set of negative controls was used on the BeadChip to estimate the background signal. A computed background signal was compared to probe signal, and a p-value was calculated. This p-value is considered as detection *p*-value. A detection *p*-value of *p* < 0.01 indicates that the measured intensity is a true signal and no background signal. Probes with detection *p*-values *p* > 0.01 were removed. Data were corrected for the type I/type II probe bias to ensure comparability of CpGs measured with different probe types (type I/type II probes). Quantile normalization with subsequent batch removal was done to reduce the technical variance from the different batches and to have equal data distribution for each sample. For batch removal the ComBat algorithm from the SVA-packages was used [27]. 

Pre-processed data was analyzed using the LIMMA package [28] within the R environment and the commercially available software Qlucore release 3.3 (https://www.qlucore.com, Qlucore Omics Explorer, Lund, Sweden) to generate the heatmaps and the plots for principle component analysis. Analysis on the single CpG level were performed in LIMMA by generating top tables with relevant information on p-values, logOddsRatio and beta-differences between groups at the single CpG level. Bumphunting from the ChAMP package was used to identify stretches with differential methylation between two groups of genomic regions with consecutive CpGs [29]. Regions with differential methylation were selected after manual inspection in the UCSC genome browser (https://genome.ucsc.edu/). Top differentially methylated CpG sites (from now on called Methylation Variable Positions (MVP)) showing hypo- and hypermethylation were defined by *p* < 0.05 and a differential methylation of β < ±0.15. Genomic regions showing differential methylation based on the selected MVPs were identified using EpiExplorer [30]. The CpG sites present on the Illumina array were used as a control set. Gene ontology (GO) term analysis of differentially methylated regions was performed using the Database for Annotation, Visualization and Integrated Discovery (DAVID). 

### 2.5. Targeted Bisulfite Sequencing

Bisulfite sequencing of top differentially methylated regions was performed by next generation sequencing using an Ion Torrent PGM platform. The MethPrimer platform (available online at http://www.urogene.org/methprimer, MethPrimer, Beijing, China) was used to design primer pairs covering the target DMRs at the promoter regions of the selected candidates. Primer design resulted in 33 assays with a length of 80–219 bp. Primer pairs were specifically designed for bisulfite sequencing PCR (BSP) to amplify the genomic regions independently of the CpG methylation status. Each primer set was tested for optimal annealing temperature and methylation bias. Each region contained between 4 and 29 CpGs (average: 10). Primers and annealing temperatures are listed in Table S2.

The respective target regions were enriched in 37 deaminated samples from the discovery cohort for internal validation and in 39 deaminated samples from the external cohort. The EZ-96 DNA Methylation Kit (Zymo Research, Irvine, CA, USA) was used for bisulfite-conversion of the samples based on the manufacture’s protocol with a starting DNA material of 400 ng. Target enrichment was done by qPCR in single PCR reactions using 10 ng of bisulfite converted DNA and with Hot Star Taq DNA polymerase (Qiagen, Cat. No. 203205) according to protocol. The polymerase chain reaction was run under the following conditions: initial activation step at 95 °C for 15 min, denaturation 1 min 94 °C, annealing 1 min (for temperature see Table S2), extension 1 min 72 °C, 40 cycles and final extension at 72 °C for 10 min, followed by pooling of the 33 targets. Library preparation and targeted sequencing for the Ion Torrent PGM was done following the manufacturer’s protocol. Briefly, after DNA end repair using the Ion Plus Fragment Library Kit (Thermo Fisher Scientific, Waltham, MA, USA), individual barcodes and sequencing adapters (Xpress Barcode Adapters, Thermo Fisher Scientific) were attached to the pooled targets of each sample and purified with AMPure XP magnetic beads (Beckman Coulter, Brea, CA, USA). Libraries were quantified using the Ion Library Quantitation Kit (Thermo Fisher Scientific, Waltham, MA, USA). Subsequently, the barcoded samples were pooled equimolar for the template preparation conducting an emulsion PCR with the Ion PGM HI-Q OT2 KIT on the Ion One Touch 2 Instrument (Thermo Fisher Scientific, Waltham, MA, USA). Enrichment of Ion Sphere Particles (ISPs) was done on the Ion One Touch ES Instrument (Thermo Fisher Scientific, Waltham, MA, USA). The quality of ISPs was controlled with the Ion Sphere Quality Control Assay (Thermo Fisher Scientific, Waltham, MA, USA) on the Qubit 2.0 Fluorometer. The 318 Chip Kit v2 (Thermo Fisher Scientific, Waltham, MA, USA) was used to sequence the sample batch with the Ion PGM Hi-Q Sequencing Kit (Thermo Fisher Scientific, Waltham, MA, USA). The sequencing reads were aligned to the hg19 reference genome and fitted to the designed amplicons. A specific workflow was used to map the reads from the Ion Torrent PGM to the reference genome [31]. 

### 2.6. Statistical Evaluation of Targeted Bisulfite Sequencing

To analyze the statistical difference between 19 responders and 18 BCG failures (internal validation cohort), we used the BRB-array tools package [32]. The methylation status of single CpG sites was calculated as the ratio of reads for methylated compared to unmethylated CpG sites multiplied by the factor 100. The following filtering set was used to pre-select the regions/samples based on the data points generated from the Targeted Bisulfite Sequencing (TBS) results: any CpG site or sample missing more than 25% of the data points generated from the Targeted Bisulfite Sequencing (TBS) was excluded from further analysis. One target region (*PTPRN2*) was excluded from further analysis due to unmet criteria. The methylation status of the differentially methylated regions was calculated as average by summing up the methylation percentage value of each single CpG site falling in the region divided by the number of CpG sites in the region. Using the BRB-Array tools, the unsupervised hierarchical clustering as well as the class comparison analysis for a p-value <0.05 were performed. To identify the best biomarker candidate, multivariate class prediction analysis for a *p*-value < 0.001 was run with the BRB-array tool program. The predictive performance of the individual DMRs was assessed by ROC curve analysis based on the average methylation status of the identified candidates. ROC curves were generated with GraphPad Prism 8 and plotted with Excel. GraphPad Prism 8 was used for graphical representation of data. 

### 2.7. Immunohistochemistry

For immunohistochemical staining, 2 µm FFPE sections were deparaffinized, rehydrated and epitopes were retrieved in 1× citrate buffer pH6 (DAKO, S1699) according to standard procedures. After incubation in 3% hydrogen peroxidase solution (Sigma Aldrich, St. Louis, MO, USA) and blocking with Serum Blocking Solution (Thermo Fischer Scientific, 859043), tissues were incubated with GPR158 antibody (Sigma Aldrich, A107248) or Paxillin antibody (Invitrogen, Carlsbad, CA, USA, AHO0492) at a dilution of 1:50 overnight at 4 degrees. Next, the tissue was washed, incubated with polyvalent biotinylated antibody (Thermo Fischer Scientific, Waltham, MA, USA, 859043) for ten minutes, stained with Enzyme conjugate (Thermo Fischer Scientific, Waltham, MA, USA, 859043) and counterstained with hematoxylin (Merck, Darmstadt, Germany, HX86017674). Pictures were taken with an Olympus Vanus AHBT3 microscope.

The evaluation of the staining intensity of the tumor area was performed by a pathologist according to the following groups: strong staining-3, medium staining-2, and weak staining-1. Staining intensity was documented for papillary regions (superficial tumor), carcinoma in situ (CIS) or invasive regions (tumor invading lamina propria) if present on the same slide, and for nuclear, cytoplasmic or vacuole localization. The final evaluation of the total staining was calculated as percentage of samples with strong, medium, or weak staining. Statistically, ANOVA-testing was used to test the significance of GPR158 or Paxillin expression among the BCG responders and BCG failures.

## 3. Results

### 3.1. Genome Wide DNA Methylation Analysis Reveals Distinct CpG Methylation Patterns for BCG Responders and Failures

**Figure 1:**
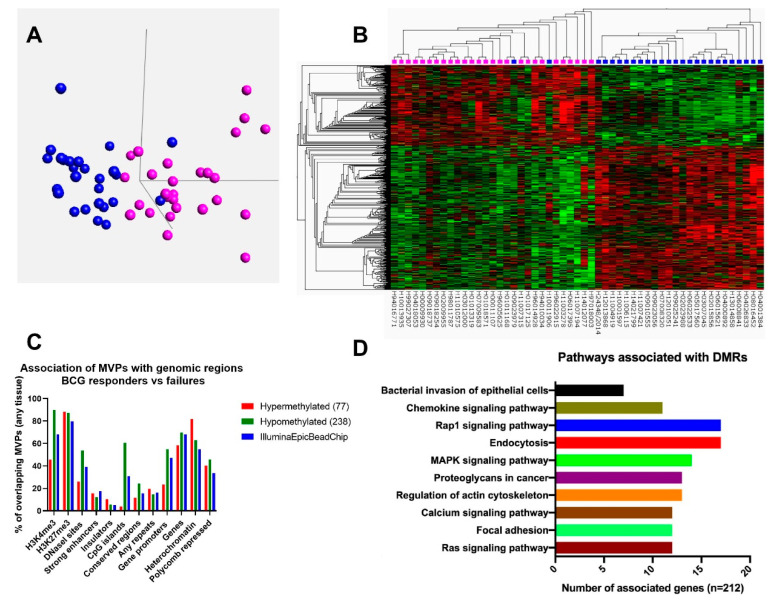
Distinct DNA methylation patterns between BCG responders and failures. (**A**) Principal component analysis of 26 BCG responders and 27 failures performed on the 15332 Methylation Variable Positions (MVPs) with a *p*-value of < 0.01 leads to grouping of the two cohorts. Sample annotation: Blue BCG responder, pink BCG failure. (**B**) Hierarchical clustering of top differentially methylated CpGs between BCG responders and failures (color corresponds to percentage of methylation, from red = 100% methylation to green = 0% methylation). Sample annotation: blue BCG responder, pink BCG failure. (**C**) EpiExplorer analysis using indicated genomic features of hypermethylated (red) and hypomethylated (green) methylation variable positions (MVPs) for BCG responders compared to BCG failures in relation to CpG sites on the Illumina array (blue). CpG sites of the Illumina array serve as control set. (**D**) Gene ontology (GO) term analysis of 694 genes harboring differentially methylated regions (DMRs) using the Database for Annotation, Visualization and Integrated Discovery (DAVID) and association with biological processes.

DNA methylation profiling of 26 BCG responders and 27 failures (discovery cohort) was performed with the Infinium Methylation EPIC BeadChip covering 850,000 CpG sites of the human genome. Clinical characteristics of patients are shown in Table 1. After data normalization and quality control, principal component analysis using v3.3 Qlucore software (Qlucore Omics Explorer, Lund, Sweden) performed on 15332 MVPs with a *p*-value of < 0.01 indicated grouping of the two patient cohorts, thus indicating that BCG responders and failures can be distinguished based on DNA methylation profiles (Figure 1A). Furthermore, two-dimensional hierarchical clustering of the top 2000 differentially methylated CpG sites (*p*-value 9.8 × 10^−4^) allowed for a clear distinction of two patient cohorts (Figure 1B). Of these MVPs, 730 (36.5%) and 1270 (63.5%) CpG sites showed decreased and increased DNA methylation in BCG responders, respectively.

### 3.2. BCG Failures Show More pronounced Gain and Loss of DNA Methylation at Sites Known for Cancer-Specific Alterations

Next, we sought to identify the genomic location of MVPs between responders and failures in the discovery cohort. For this, only MVPs with a potential biological significant difference in methylation were considered. For selection, we applied a *p*-value < 0.05 and a beta-value difference of ± 0.15 (representing at least a 15% difference in methylation) as filtering criteria. Among all identified MVPs, 315 CpG sites met these criteria, of which 75% showed lower methylation in responders compared to BCG failures (Table S3). To investigate the genomic distribution of these 315 MVPs, we used the EpiExplorer web based tool, which associates given CpG sites with genomic regions and features of publicly available datasets [30]. The overall Illumina CpG annotation served as a control set. As depicted in Figure 1C, 60% of the CpG sites hypomethylated in responders overlapped with CpG islands, 58% with gene promoters and 55% with DNAseI hypersensitive sites. However, only 30.9%, 47.2% and 39.3% of the control set overlapped with these regions, respectively. Further, hypermethylated CpG sites in responders compared to failures were over-represented at heterochromatic regions compared to the control set.

### 3.3. Differentially Methylated Regions between BCG Responders and Failures are Associated with Genes Involved in Bacterial Uptake and Cell-Adhesion Pathways

To identify pathways and biological processes that might be affected by differential methylation, we sought to identify genomic regions containing multiple MVPs. Differentially methylated regions (DMRs) were defined by the bumphunting method from the Champ package as described in the methods section. Overall, 694 genes were identified, which contained DMRs with an adjusted *p*-value < 0.05 (Table S4). To test the association of these 694 genes with biological processes, Gene ontology (GO) term analysis using the Database for Annotation, Visualization, and Integrated Discovery (DAVID) was performed. DAVID showed an association with biological processes such as bacterial invasion of epithelial cells, chemokine signaling, endocytosis, focal adhesion and the MAPK or RAS signaling pathways [33,34] (Figure 1D). In order to investigate a potential biological mechanism differing between responders and failures, we also evaluated protein expression of selected genes harboring DMRs belonging to these pathways, such as paxillin, which is a focal adhesion protein involved in bacterial uptake [35,36,37]. However, although DNA methylation in the *PXN* promoter and gene body was lower in BCG responders than failures, we did not find significant differences in protein expression between the two cohorts. Still, there was a trend towards slightly stronger expression in the responders’ cohort (Figure S2A–C).

### 3.4. Identification of Candidate DNA Methylation Biomarkers for BCG Response

**Figure 2:**
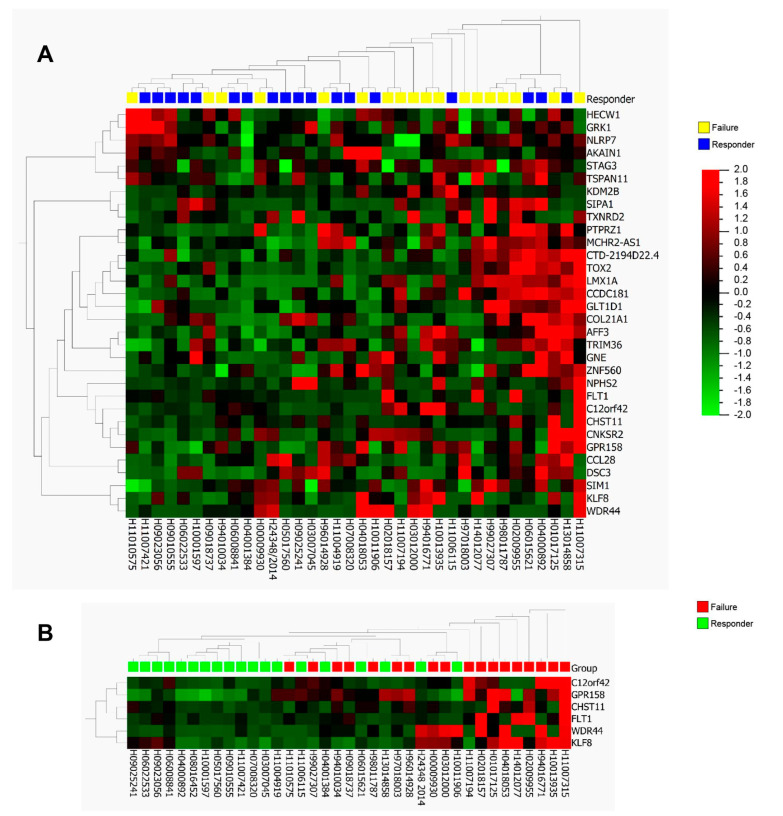
Bisulfite-sequencing (BSP) of identified top DMRs between BCG responders and failures using an internal validation cohort. (**A**) Hierarchical clustering of the BSP results of selected top DMRs results in separate clusters for BCG failures and responders (color corresponds to percentage of methylation, from red = 100% methylation to green = 0% methylation). (**B**) Six target regions identified with class comparison analysis in the promoters of *GPR158*, *KLF8*, *C12orf42*, *WDR44*, *FLT1,* and *CHST11* with a significantly differential methylation status between the two patient cohorts.

**Figure 3:**
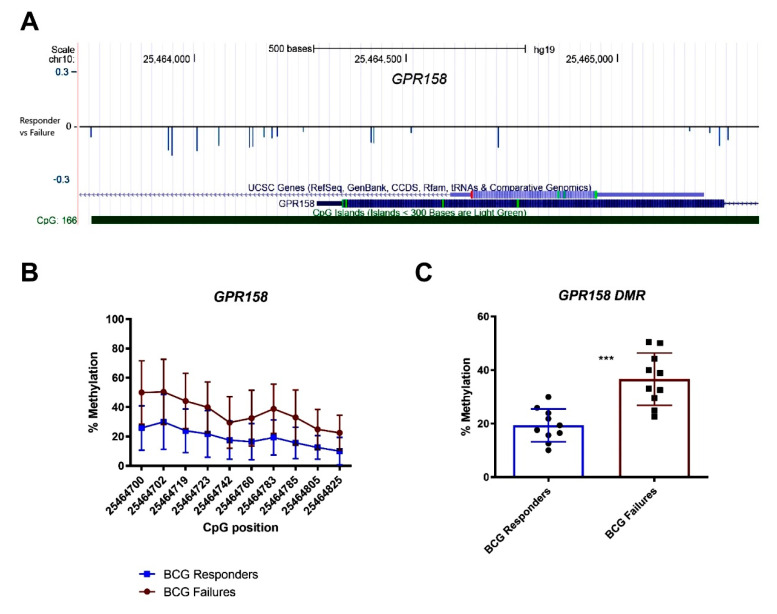
DMR in *GPR158* gene promoter as example for differential methylation between BCG responders and failures. (**A**) Graphical representation of the DMR located in the *GPR158* promoter. (**B**) Bisulfite sequencing (BSP) results of single CpGs within in the *GPR158* promoter shown as percentage of methylation and (**C**) comparison of overall DNA methylation percentage differences of DMR in *GRP158* promoter between cohorts (blue BCG responders, red BCG failures).***: *p*-value < 0.001.

**Figure 4:**
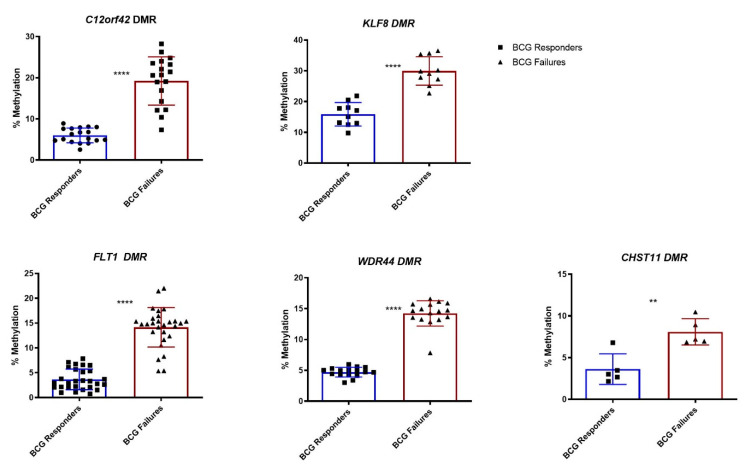
Comparison of overall DNA methylation percentage differences of the DMRs in the promoters of *KLF8*, *C12orf42*, *WDR44*, *FLT1,* and *CHST11*. Error bars indicate mean ± SD. Note that individual values represent average DNA methylation values of respective CpG sites within the promoters. **: *p*-value < 0.01, ****: *p*-value < 0.0001.

For validation, we selected 33 DMRs located at promoter regions and performed bisulfite-sequencing of these regions (internal validation cohort *n* = 37; responders = 19, failures = 18) (Tables S1 and S2). In order to identify target regions (DMRs) for potential clinical use as predictive biomarkers for BCG response, the average methylation level for each target region was calculated. Unsupervised hierarchical clustering of the BSP results of the selected 33 regions gave separate clusters for BCG failures and responders (Figure 2A). Class comparison analysis identified six target regions with a significantly differential methylation status between the two patient cohorts. These regions were located in the promoters of *GPR158* (10 CpG sites), *KLF8* (10 CpG sites), *C12orf42* (18 CpG sites), *WDR44* (16 CpG sites), *FLT1* (28 CpG sites), and *CHST11* (5 CpG sites) (Figure 2B). A graphical representation of the DMR located in the *GPR158* promoter CpG island is shown as an example (Figure 3A). Bisulfite sequencing (BSP) of the region was performed to correlate the DNA methylation differences with those obtained by genome wide methylation analysis (Figure 3B,C). A difference of approximately 20% DNA methylation was present between samples from BCG responders and failures in this region, with increased DNA methylation in the failures. BSP results of the DMRs in the promoters of *KLF8*, *C12orf42*, *WDR44*, *FLT1,* and *CHST11* are shown in Figure 4. Differential methylation of all six regions was also validated in an external cohort of 39 patients (Figure S3). 

### 3.5. GRP158 Shows a Higher Degree of Methylation in Patients with BCG Failure

**Figure 5:**
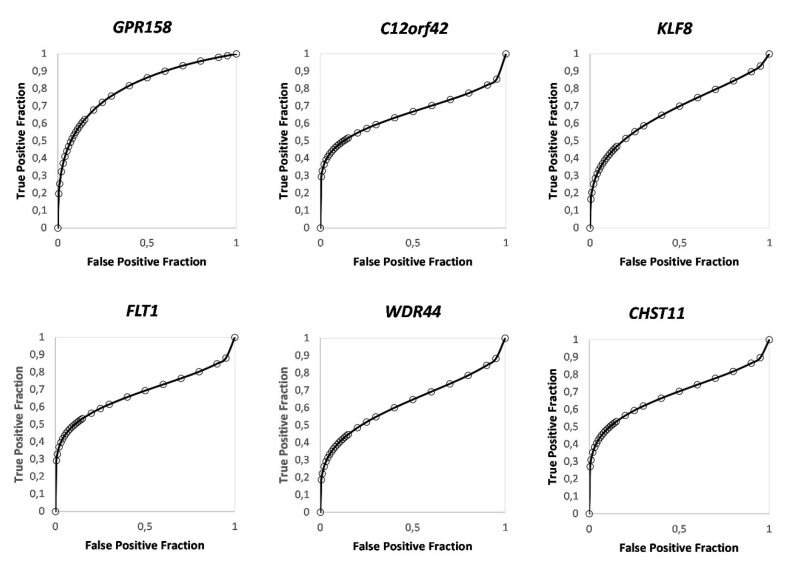
Receiver operator curves (ROC) and areas under the curve (AUCs) for the six DMRs.

To further test the biomarker potential of the candidate regions, multivariate class prediction analysis using the BRB-array tools was performed. Using a stringency with a *p*-value of <0.001, the promoter region of *GPR158* resulted as best predictor with an area under the curve (AUC) of 0.809 (Figure 5). Receiver operator curves (ROC) for the other regions resulted in AUCs between 0.633 to 0.681 (Figure 5). ROC curves for the external validation cohort are shown in Figure S4. 

Next, we investigated a potential correlation of DNA methylation of the *GPR158* promoter region with protein expression. Staining of tumor tissues of 14 BCG responders and 18 failures with a GPR158-specific antibody was performed; however, tumors of both cohorts displayed varying degrees of GPR158 protein expression. Consequently, no statistically different expression between the two groups was observed (Figure S5A,B).

## 4. Discussion

After BCG therapy, approximately 40% of bladder cancer patients present with disease recurrence within 5 years, while 20% progress to MIBC [38]. Even though clinical trials on various systemic treatment are presently ongoing, radical cystectomy is the first treatment option after BCG failure. Thus, the development of novel biomarkers to predict BCG failure are necessary to avoid ineffective therapy and allow for a personalized therapy. Here, we investigated the genome-wide DNA methylation profile of tumors from BCG responders and failures to get an insight into epigenetic differences, thereby identifying DNA methylation based biomarker candidates. 

In our dataset of 26 BCG responders and 27 failures, we identified 2000 CpG sites with significant differential methylation, allowing a clear distinction of the two groups. Noteworthy, two patients belonging to the responder cohort incorrectly grouped with the failures (Figure 1A,B). However, one of these patients had a recurrence albeit of a pTa low grade tumor within six month of therapy start.

The genomic regions associated with the most pronounced DNA methylation differences between BCG responders and failures were predominantly located at CpG islands, gene promoters and heterochromatin. CpG islands and gene promoters are genomic regions that usually gain DNA methylation in the transition from healthy to malignant tissue, whereas heterochromatic regions lose DNA methylation in cancer. Indeed, we found increased DNA methylation of CpG islands and gene promoters in BCG failures compared to responders, whereas the inverse effect was observed for differences found at heterochromatin (Figure 1C). Given this overlap with regions that usually show cancer-specific gain or loss during the transition from healthy to malignant state, we hypothesize that an increase in DNA methylation at CpG islands or gene promoters and a loss at heterochromatin in BCG failures might indicate a more de-differentiated state, suggesting a more aggressive cancer phenotype. In line with our hypothesis, studies in different tumor entities have revealed an increase in the difference of methylation with advancing tumor stage [39,40]. In bladder cancer, genome wide analyses of CpG islands have revealed a higher degree of methylation in high grade compared to low grade tumors [41]. Furthermore, molecular analysis of aggressive and more advanced epithelial tumors have shown that stem cell signatures both at genetic and epigenetic level increase with tumor dedifferentiation [42]. 

Besides differences in DNA methylation at distinct genomic regions, we also detected differentially methylated CpG sites in genes involved in specific cellular pathways including bacterial uptake by epithelial cells, endocytosis, focal adhesion and Ras signaling. Mechanistically, BCG action is thought to occur by BCG attachment to and internalization by urothelial cancer cells, and a lack of BCG uptake is associated with reduced immunologic responses to treatment [11,12,13,14,15,16,17,18,19,20]. In addition, activation of RAS has been shown to be advantageous in BCG uptake [14]. After uptake, BCG is presented to immune cells including CD4+ and CD8+ lymphocytes, natural killer cells, granulocytes, macrophages and dendritic cells. The cancer cells are eventually killed through direct and indirect cytotoxic effects. Secretion of soluble factors such as tumor necrosis factor-related apoptosis-inducing ligand and a limited direct effect can also contribute to BCG’s effect [8,10]. Thus, epigenetic modifications of genes involved in adhesion and bacterial uptake of cells may be a molecular feature distinguishing BCG responders and failures. Protein products of several of the genes affected by altered DNA methylation have already been associated with bladder tumor aggressiveness. For example, in vitro studies have shown that paxillin, a protein involved in focal cell adhesion and bacterial uptake, is involved in cell migration and invasion [35,36,37,43]. It has also been reported that bladder tumors of more advanced stages express lower levels of paxillin [44]. In our study, *PXN* coding for paxillin was among the genes identified to harbor DMRs (Table S4), whereby protein expression did not show significant differences between the two cohorts (Figure S2) [45]. Still, tumors with the highest level of paxillin expression were present to a larger extend in the group of BCG responders, suggesting that our relatively small cohorts lack the power to investigate minor differences in protein expression. 

Regarding potential biomarkers to differentiate between BCG responders and failures, we focused on genomic regions such as CpG islands rather than single CpG sites as these may show correlation with biologic activity and altered gene expression. We identified six DMRs located in the promoters of *GPR158*, *KLF8*, *C12orf42*, *WDR44*, *FLT1,* and *CHST11*, among which *GPR158* promoter methylation showed the best performance (AUC = 0.809). GPR158 is a G-protein coupled receptor of the GPCR family C located at the plasma membrane and in the nucleus. It is known to be involved in neuronal differentiation and neuronal signaling pathways [46,47]. Regarding the genitourinary system, it has been shown to stimulate prostate cancer growth and progression [48], but its role in bladder cancer was not described before. In our study, protein expression levels were not significantly different between the groups, which may be explained by the low number of samples stained. 

Predictive DNA methylation biomarkers for BCG response have been reported by several publications [22,23,49,50]. Among the reported biomarker candidates, the respective regions in the genes of *HOXA9*, *PRMD14*, *NKX6-*2, and *SPAG6* were differentially methylated in our cohorts, albeit not among the top candidates and with lower overall DNA methylation differences (Table S4 and data not shown). This might be due to different definitions of BCG failure as well as different study designs. For example, while Kitchen et al. considered methylation differences of more than 10% as significant in their study, we set a threshold of more than 15%, which might result in the inclusion of different CpG sites for further analysis. Secondly, in our study we have used the Illumina EpicBead Chip, which interrogates a higher number of CpG sites in comparison to the Illumina 450K used by Kitchen et al. Finally, our study focused on the identification of differentially methylated genomic regions rather than of differences at single CpG sites [22]. 

Noteworthy, we want to highlight that all six top DMRs in our study already show a certain degree of DNA methylation in BCG responders, and although a significant increase in DNA methylation from responders to failures is observed in our samples, a threshold value as cut off for distinguishing between the two groups is difficult to set. Thus, biomarkers for clinical routine based merely on DNA methylation do not seem feasible in routine diagnostics, as DNA methylation levels between responders and failures at single CpG sites or regions may not differ sufficiently for adequate separation. 

Our study has several limitations. First, we defined patients as unresponsive to BCG, when showing progression within three to six months or persistent high-grade disease at six months after starting therapy. We further included patients with tumor relapse within six months of last BCG exposure in this group, and patients with early or late recurrences (after six months of last BCG exposure). Although the majority of patients were BCG refractory and early relapse patients, the cohort is heterogeneous regarding the time point of BCG failure, and different molecular mechanisms may contribute to BCG failure at different time points. Furthermore, for patients classified as BCG failures, it is a possibility that their primary tumor may initially not have been completely resected during TURB and second-look TURB. This, in turn, leads to subsequent therapy failure, but also wrong classification as “BCG failures”.

Regarding the coverage of genome-wide methylation analysis, the platform we were using interrogates 850,000 CpG sites of the human genome. Although the vast majority of CpG islands, genes, gene promoters, and enhancers are covered by this method, approximately 97% of the CpG sites in the human genome are not analyzed and may include differences we were not able to assess [51]. Also, we report findings from a relatively small sample size, which may not allow the evaluation of modest DNA methylation differences or minor differences in protein expression between BCG responders and failures. Finally, in light of clinical applicability, future studies investigating DNA methylation differences may focus on obtaining liquid biopsies directly from urine or blood, as this would simplify sample collection and analysis time. 

## 5. Conclusions

Genome-wide DNA methylation profiling is able to differentiate BCG responders from failures, and processes like bacterial uptake, endocytosis, or focal adhesion are affected by differential methylation. *GPR158* seems to be an interesting promoter associated with a higher rate of DNA methylation in BCG failure patients. Nevertheless, this difference in DNA methylation did not translate to protein expression. Further studies are needed to test the biomarker potential of DNA methylation differences between BCG responders and failures and correlate *GPR158* DNA methylation with protein expression as well as potential clinical applicability.

## Figures and Tables

**Table 1:** Clinicopathological characteristics of Bacillus Calmette-Guérin (BCG) patients for Illumina array analysis.

Characteristic ^1^	Responders (*n* = 26)	Failures (*n* = 27)
**Age**, y		
Median	69	70
Range	51–86	54–93
**Sex**, no. (%)		
Male	23 (88)	20 (74)
Female	3 (12)	7 (26)
**T stage**, no. (%)		
Tis	0	1 (4)
Ta	7 (27)	2 (8)
T1	19 (73)	24 (88)
T1a	10 (38)	8 (30)
T1b	6 (23)	4 (14)
T1 ns	3 (12)	12 (44)
**Grade**, no. (%)		
High	24 (92)	24 (89)
Not specified	2 (8)	3 (11)
**Concomitant CIS**, no. (%)	16 (62)	11 (41)
**Type of failure**, no. (%)		
BCG refr. + early rec.	-	18 (67)
BCG late rec.	-	9 (33)
**Follow-up since TURB**, m	26 (5–109)	12 (2–38)

^1^ CIS: carcinoma in situ; m: months; no.: number; ns: not specified; rec.: recurrence; refr.: refractory; T: tumor; TURB: transurethral resection of the bladder; y: years.

## Supplementary Materials

The following are available online at https://www.mdpi.com/2073-4409/9/8/1839/s1, Figure S1 Definition of BCG failure. Figure S2 *PXN* genomic region and paxillin protein expression. A *PXN* genomic region with decreased DNA methylation in promoter and gene bodies of BCG responders. B No significant difference in staining intensity is detected between BCG responders and failures. C Representative IHC staining of paxillin for each cohort. Figure S3 BSP results and DNA methylation differences in the external validation cohort of the six DMRs. Figure S4 ROC curves and AUC of the six DMRs in the external validation cohort. Figure S5 GPR158 protein expression in BCG responders and failures. A No significant difference in staining intensity is detected between BCG responders and failures. B Representative IHC staining of GPR158 for each cohort. Table S1 Clinicopathological characteristics of BCG patients (internal and external cohort) for validation analysis. Table S2 Primer pairs and annealing temperatures for bisulfite sequencing of top DMRs. Table S3 List of significantly hypo- and hypermethylated CpG sites between BCG responders and failures. Table S4 List of top DMRs between BCG responders and failures.
