# Supplementary figures and images for: Discovery of Molecular DNA Methylation-Based Biomarkers through Genome-Wide Analysis of Response Patterns to BCG for Bladder Cancer
Source: Cells. 2020 Aug 5;9(8):1839. doi: 10.3390/cells9081839 (PMC7464079; doi:10.3390/cells9081839)

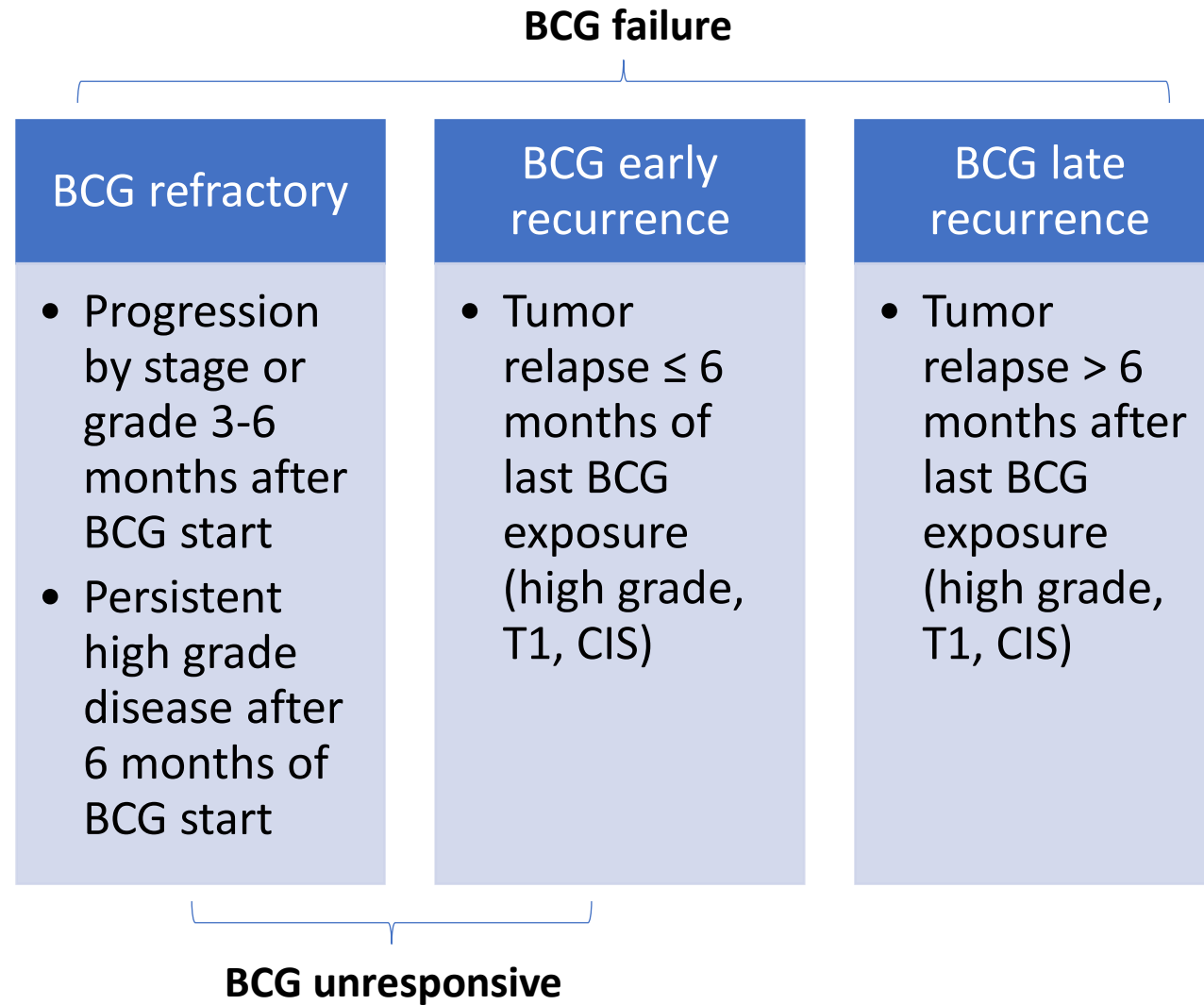

Supplementary Figure 1

Supplement: Supplementary file 1 [file cells-09-01839-s001.zip › Supplementary Figure1.pdf]

**A**

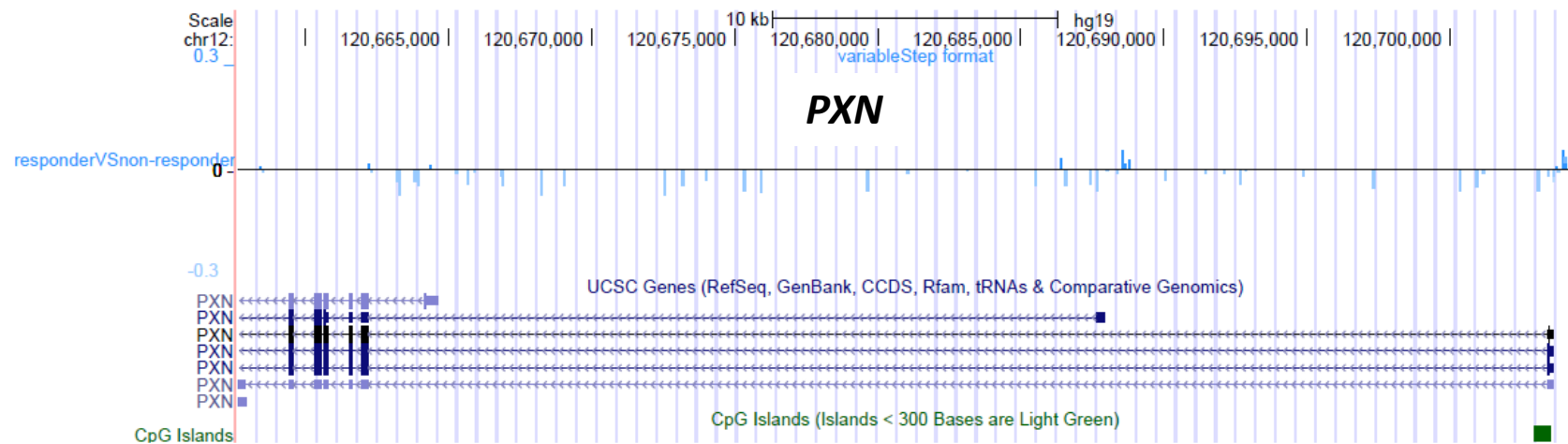

Supplementary Figure 2

**B Paxillin protein expression**

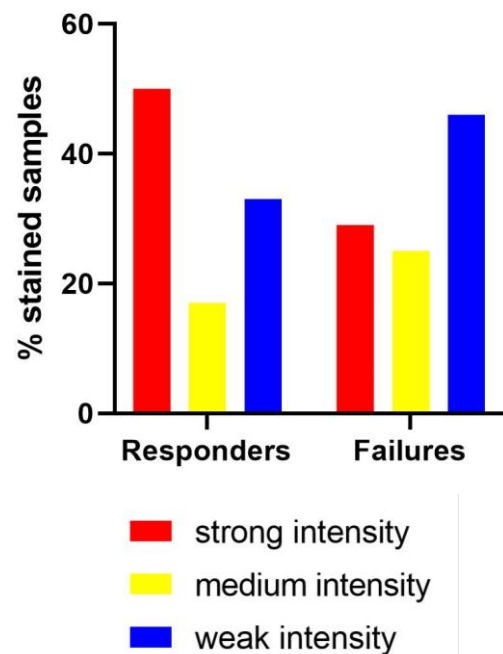

**C**

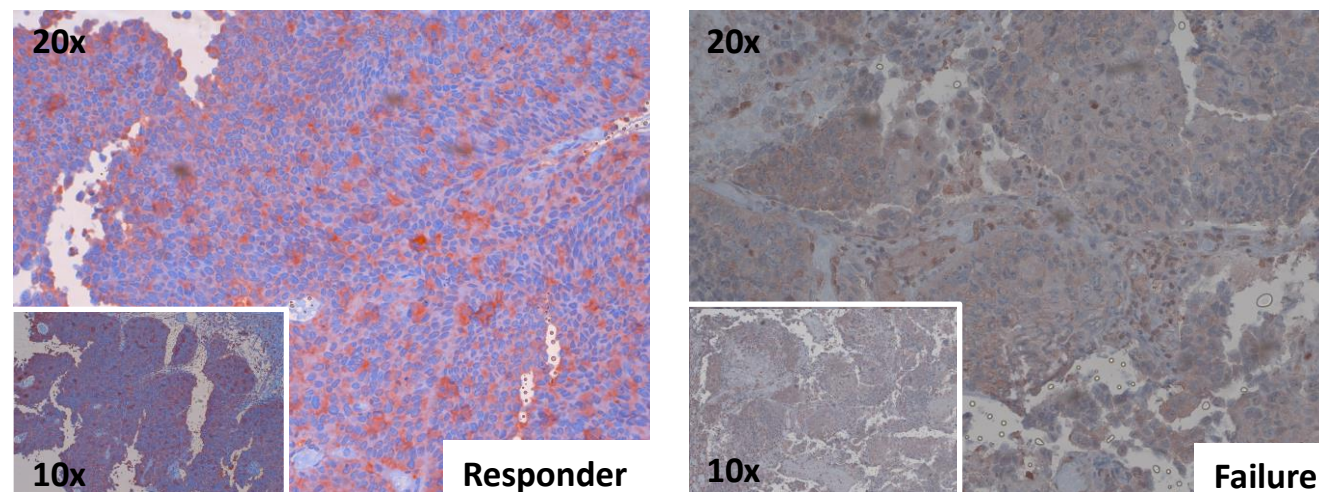

Supplement: Supplementary file 1 [file cells-09-01839-s001.zip › Supplementary Figure2.pdf]

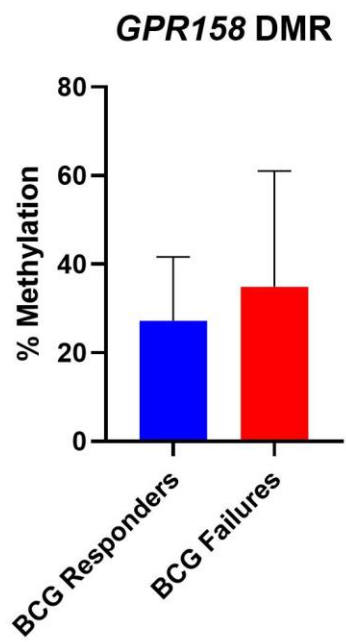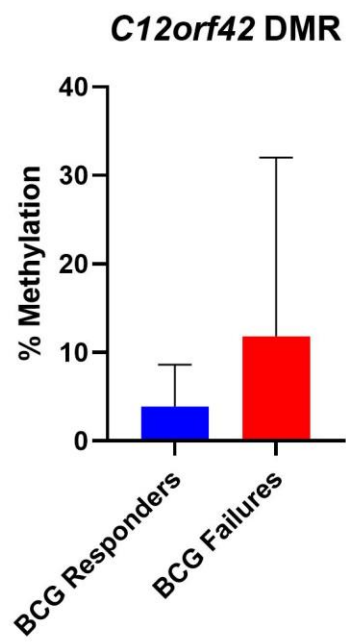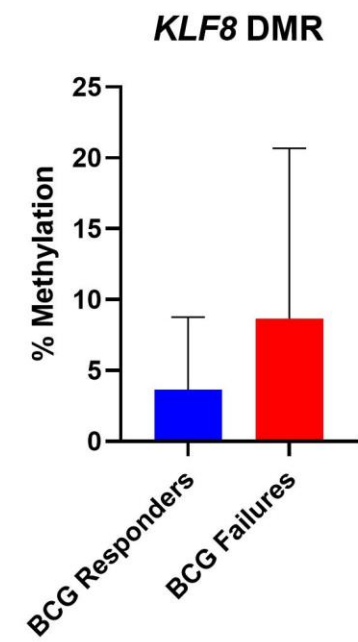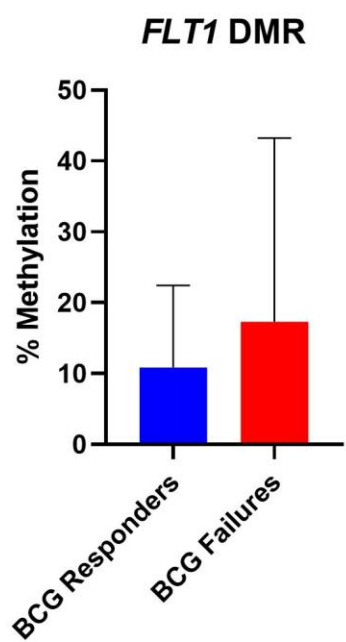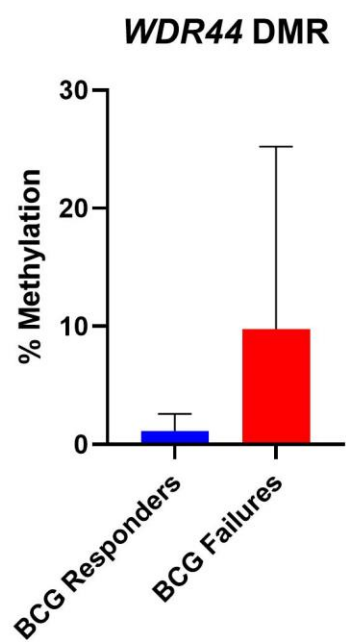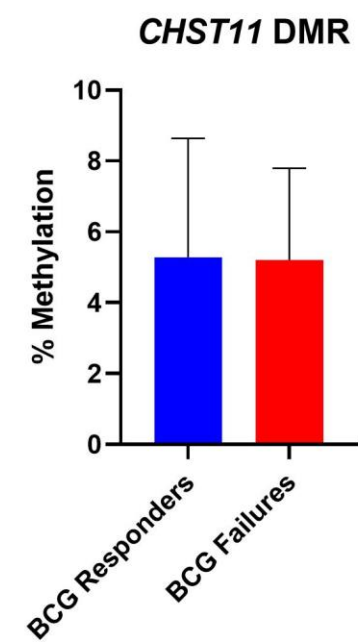

Supplementary Figure 3

Supplement: Supplementary file 1 [file cells-09-01839-s001.zip › Supplementary Figure3.pdf]

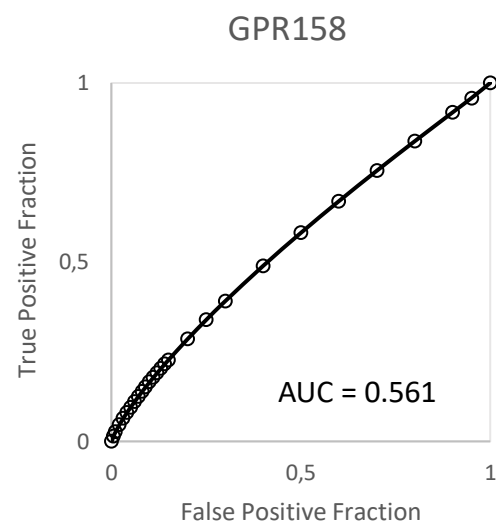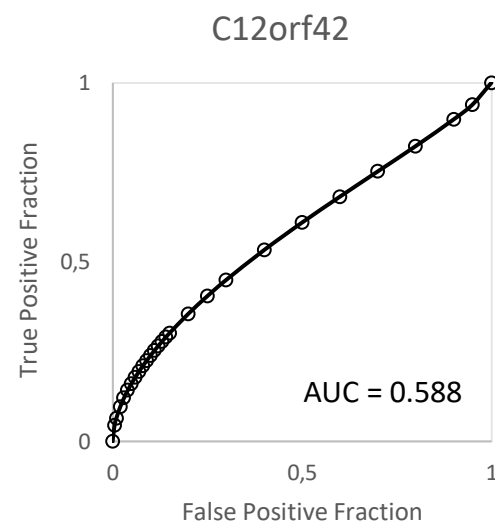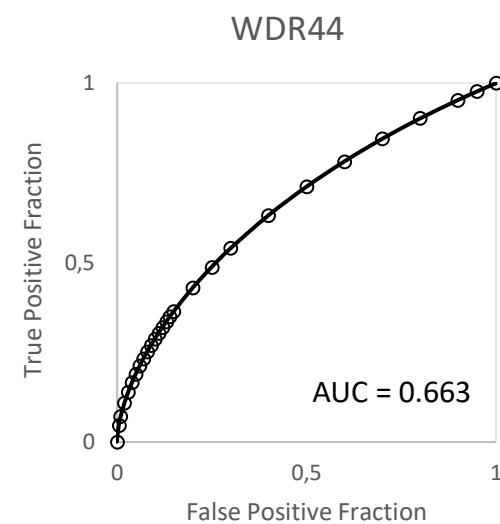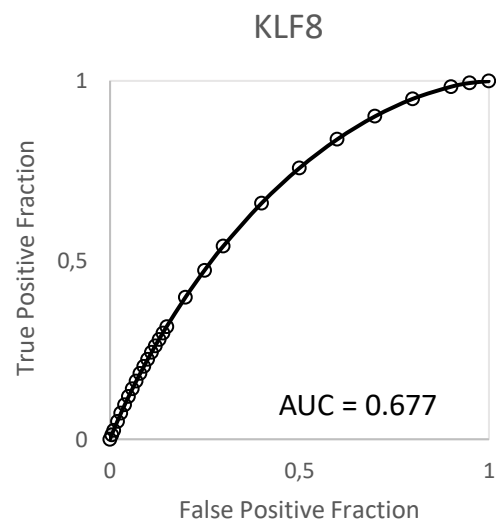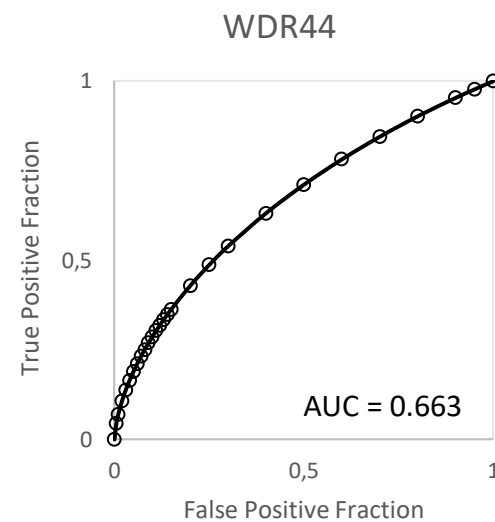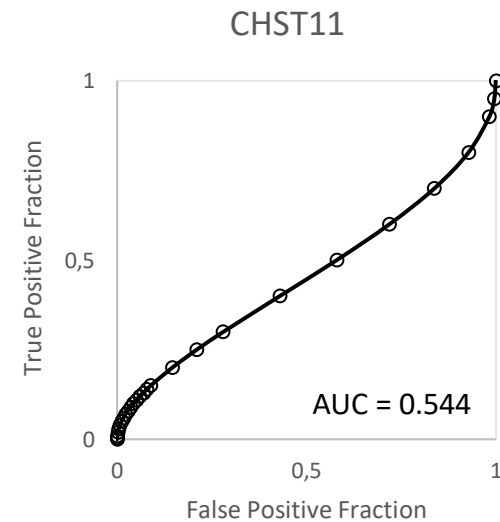

Supplementary Figure 4

Supplement: Supplementary file 1 [file cells-09-01839-s001.zip › Supplementary Figure4.pdf]

## A GPR158 protein expression

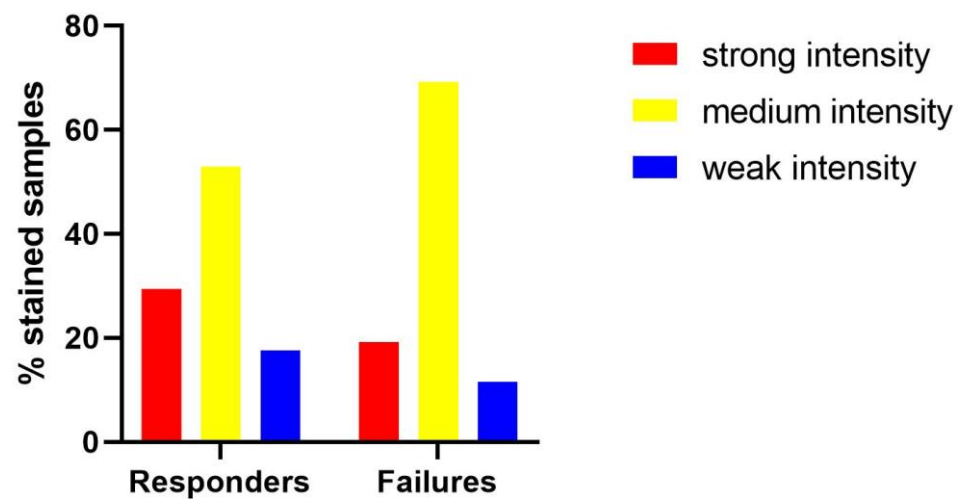

Supplementary Figure 5

## B

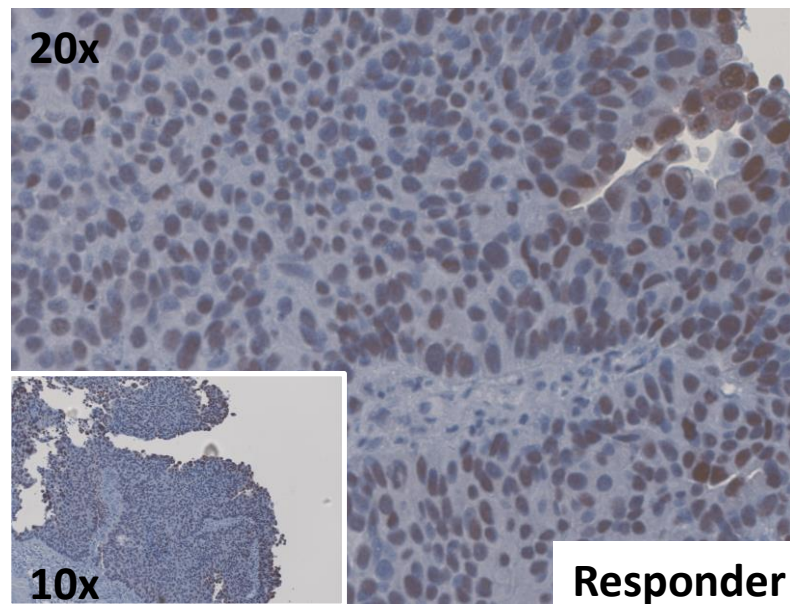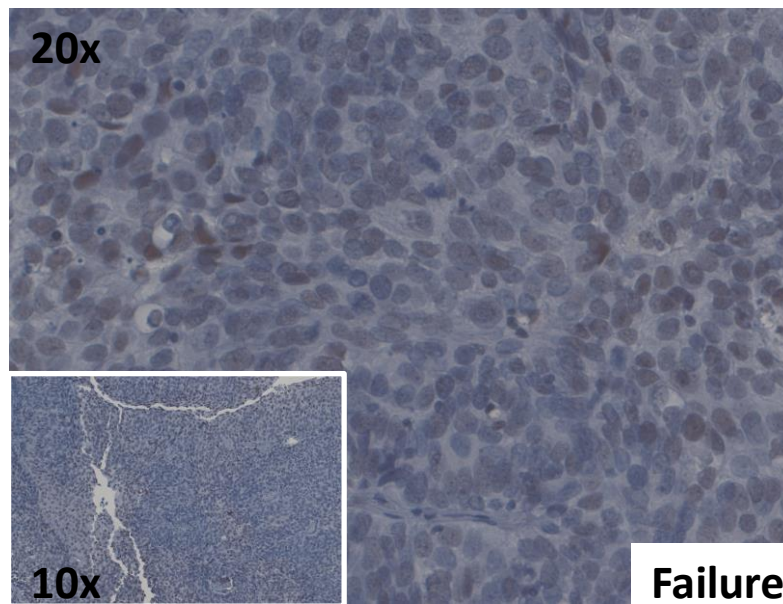

Supplement: Supplementary file 1 [file cells-09-01839-s001.zip › Supplementary Figure5.pdf]
